# The Effect of Neurofeedback on the Reaction Time and Cognitive Performance of Athletes: A Systematic Review and Meta-Analysis

**DOI:** 10.3389/fnhum.2022.868450

**Published:** 2022-06-20

**Authors:** Michele Andrade de Brito, José Raimundo Fernandes, Natã Sant'Anna Esteves, Vanessa Teixeira Müller, Daniella Brito Alexandria, Diego Ignacio Valenzuela Pérez, Maamer Slimani, Ciro José Brito, Nicola Luigi Bragazzi, Bianca Miarka

**Affiliations:** ^1^Laboratory of Psychophysiology and Performance in Sports & Combats, Department of Physical Education, Federal University of Rio de Janeiro, Rio de Janeiro, Brazil; ^2^Escuela de Kinesiología, Facultad de Salud, Magister en Ciencias la Actividad Física y Deportes Aplicadas al Entrenamiento Rehabilitación y Reintegro Deportivo, Universidad Santo Tomás, Santiago, Chile; ^3^Department of Neuroscience, Rehabilitation, Ophthalmology, Genetics, Child and Maternal Health, Faculty of Medical and Pharmaceutical Sciences, University of Genoa, Genoa, Italy; ^4^Department of Physical Education, Federal University of Juiz de Fora, Campus Governador Valadares, Governador Valadares, Brazil; ^5^Laboratory for Industrial and Applied Mathematics, Department of Mathematics and Statistics, York University, Toronto, ON, Canada

**Keywords:** performance, cognitive training, executive functions, sport psychology, brain stimulation

## Abstract

Our study evaluated the effect of training with neurofeedback (NFB) in improving athletes' reaction time and decision-making. A computerized search in PubMed, PsycINFO, Scielo, Web of Science, EMBASE, Scopus, BVS, and Cochrane databases was performed to identify studies published from 2011 to June 2021. The protocol was registered in PROSPERO. The quality of studies that was peer-reviewed and included was assessed using the Review Manager tool, Cochrane Risk of Bias, and design and reporting quality according to the CRED-nf checklist. Standard mean differences and 95% confidence intervals (CIs) were calculated and combined using a random-effects model. A total of 07 randomized controlled trials (RCTs) (173 athletes) met the inclusion criteria. Significant effects of NFB in the experimental group in relation to reaction time were found, indicating an improvement in sports performance [standardized mean difference (SMD) = −1.08; 95% CI = (−1.90, −0.25), *p* = 0.0009] and cognitive performance vs. decision-making with moderate effect [SMD = 1.12; 95% CI = (−0.40, 1.85), *p* = 0.0001]. However, the control group had a very small effect on cognitive performance [SMD = 0.19; 95% CI = (−0.20, 0.59), *p* = 0.086]. NFB could improve athletes' reaction time and decision-making, effectively increasing their performance in the sports field. Future studies should focus on standardized protocols for NFB training.

**Systematic Review Registration:**

http://www.crd.york.ac.uk/PROSPERO/, identifier: CRD42021258387.

## Introduction

Experienced athletes show a consistent performance at optimal levels (Filho et al., 2021), and all performance situations in sports require reaction time (Mirifar et al., 2017) and cognitive skills (Liu et al., 2017), so it is necessary to look for adequate and effective training protocols in the literature. There is a consensus that meta-analysis is at the top of the evidence in the scientific pyramid. It is an appropriate statistical technique to combine the results from different studies (Berwanger et al., 2007), being an essential source of evidence to assist in decision-making regarding interventions in sports, such as neurofeedback (NFB). Furthermore, the improvement of health, associated with the athletes' performance, is a primary concern, since technical–tactical skills, physical, and mental fitness form the triad of sports development (Gomes and Rêgo, 2019). Despite the existence of this triad, there is an exacerbated concern with physical fitness, while technical–tactical and mental skills are secondary due to misconceptions that the limit is the plateau of physical capabilities (Diamond, 2013; Diamond and Ling, 2016). Based on this paradigm, it is essential to explore the methods of cognitive development that reduce physical demands and improve specific skills, with more effective actions, through the improvement of cognitive performance (e.g., attention, concentration, memory, inhibitory control, cognitive flexibility, and focus) and time to reaction (response time), an example of this is NFB.

The NFB is a relatively novel method to improve the performance of athletes (Rostami et al., 2012). This technological resource of self-regulatory stimulation is used to rebalance brain-functioning patterns to improve cognitive, emotional, and behavioral performance (Salimnejad et al., 2019; Gong et al., 2021). NFB uses measured changes in brain activation to help athletes regulate the activity (in particular, regions or networks) or power of designated EEG frequency bands by providing them with the activation information in real time. Therefore, during NFB training, participants are trained to self-regulate their brain activity, usually with the ultimate goal of changing behavioral or cognitive/emotional functions (Sitaram et al., 2017; Paret et al., 2019). In this study, we will emphasize the use of NFB, reaction time, and cognitive performance indices of athletes.

Reaction time or response time refers to the amount of time it takes from the moment we perceive something to when we respond to the stimulus, and it is the ability to detect, process, and respond to a stimulus (Mirifar et al., 2019). It has been an index that allows the assessment of internal cognitive-motor resources associated with the athlete's performance (Parsaee et al., 2018; Mirifar et al., 2019), significantly inferring the individual's ability to make complex decisions and initiate actions quickly and effectively (Araújo et al., 2019). The study by Parsaee et al. (2018) investigated the effect of NFB training on visual and auditory reaction time and concluded that NFB effectively improves brain functions for visual and auditory reaction time.

Another index that we can use to assess the cognitive performance of athletes that seems to be congruent with most sports is executive functions, i.e., a set of goal-oriented cognitive processes that allow us to control and regulate our thoughts, emotions, and actions in the face of adversity (Diamond, 2013; Russo and Ottoboni, 2019). According to the study by Liu et al. (2017), NFB training proved to help improve cognitive skills for athletes, the efficiency of NFB training was examined by comparing shooting scores, and Sustained Attention Test (DAUF) test results evaluated the sustained attention capacity of athletes (shooters) before and after NFB training.

These processes can be improved through NFB training (Mikicin et al., 2015; Mirifar et al., 2017, 2019; Salimnejad et al., 2019). The critical characteristics of optimal performance include good reaction time levels and mental abilities (Blumenstein and Orbach, 2020). Thus, NFB training can significantly improve athletes' reaction time (Mikicin et al., 2018) and cognitive performance (Schönenberg et al., 2017; Crivelli et al., 2019). However, there are still gaps to be filled out on the inference of the protocols on the effect size on the performance of athletes (Mirifar et al., 2017; Xiang et al., 2018). The study aims to evaluate the effect of NFB on the reaction time and cognitive performance of athletes.

## Methods

The study registered the protocol in PROSPERO and can be found in https://www.crd.york.ac.uk/PROSPERO/#recordDetails. Data are available in the Open Science Framework *via* the link (Page et al., 2018). We adhered to the PRISMA guidelines for organizing systematic reviews and meta-analyses (Tricco et al., 2018). We focused our research on athletic athletes.

### Research Strategy

This study used the following electronic bibliographic databases for studies published until 7 July 2021: PubMed, PsycINFO, Scielo, Web of Science, EMBASE, Scopus, BVS, and Cochrane. The descriptors of the respective Medical Subject Headings (MeSH) Descriptors in Health Sciences (DeCS) were consulted and combined with the Boolean operators AND and OR. The following search terms were used: NFB, time reaction, decision-making, and sports. Filters were used for the years 2011–2021, randomized and controlled studies. More details on the search strategy can be found in the protocol or at the following link.

### Selection of Studies

The first two authors conducted the research independently. The EndNote software was used to screen the articles (Bramer et al., 2017); with a reading of titles and abstracts, we included all published articles with study designs that applied NFB training to regulate brain activity and/or behavior in sports participants.

**Figure 1:**
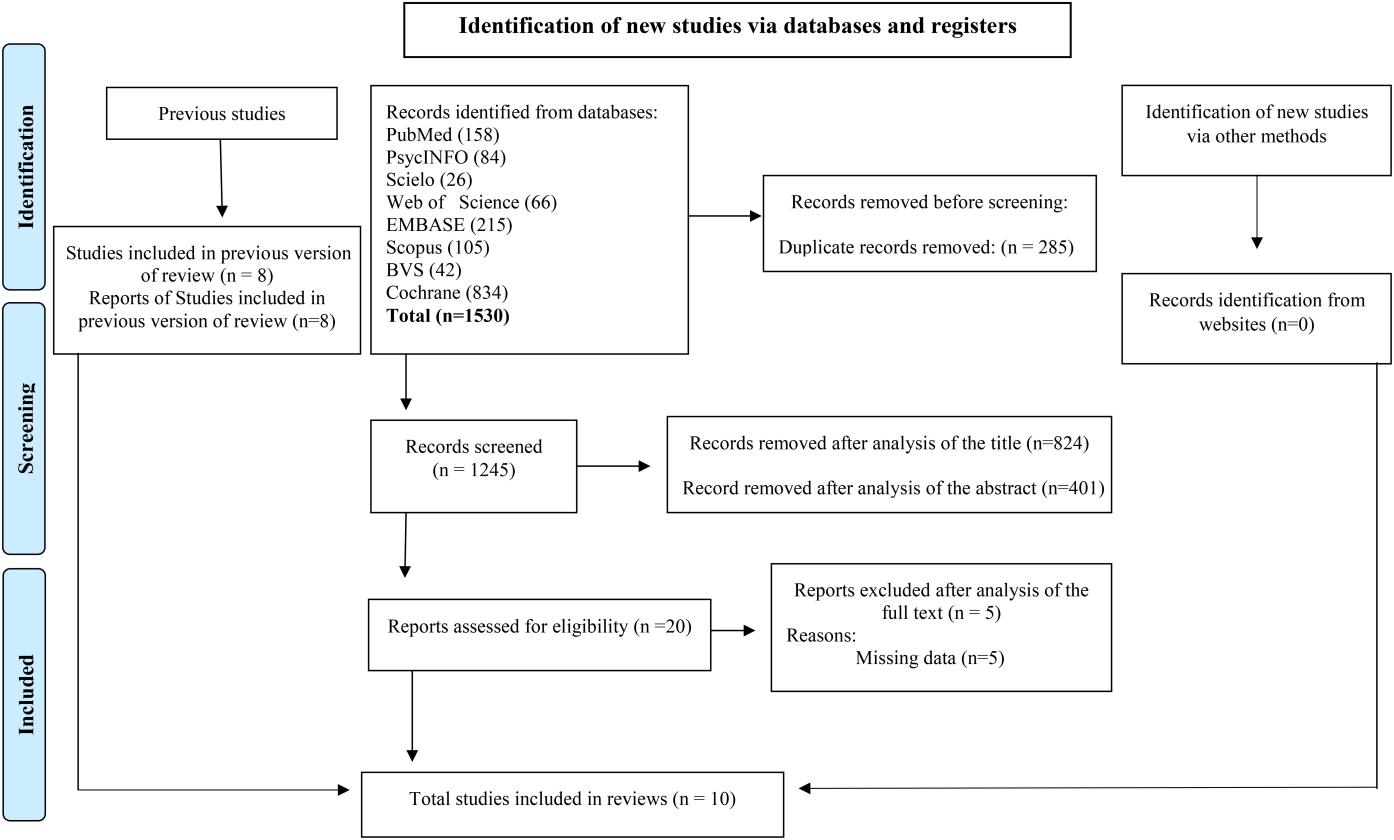
PRISMA flow diagram for study selection.

In the next step, duplicates were removed, articles were read, and those that did not have enough information to apply the eligibility criteria were excluded; for this step, we used the Rayyan software (Ouzzani et al., 2016). After the evaluation, the two authors plus a judge (third author) met to reach a consensus regarding the inclusion and exclusion criteria for each article, and disagreements were judged and a consensus was reached for all included articles. The agreement between raters was 97%. The description of the selection of studies is shown in Figure 1.

### Data Extraction and Analysis

For articles that met the eligibility criteria, we extracted the following information for qualitative analysis:

- authors and year of publication;
- participant characteristics (e.g., sample number, age, gender, type of sport, and time of sport);
- characteristics of the studies (e.g., intervention, type of protocol used, evaluation and measurements performed, training of brain regions, electrodeposition, waves, and frequencies trained) and the outcomes.

The groups (NFB training modalities) will be compared with the quantitative analysis. In all included studies, summary outcome statistics were means (M) and standard deviation (SD). After the completion of data extraction, the spreadsheet was sent to all corresponding study authors for corrections. All authors approved data extraction or submitted minor corrections. The qualitative analysis is detailed in Tables 1, 2.

**Table 1:** Consensus assessment on the reporting and experimental design of clinical and cognitive-behavioral neurofeedback studies (CRED—NF).

	**CRED_NF**																						
**References | Checklist**	**1. Pre-experiment**		**2. Control Groups**					**3. Control Measures**					**4. Feedback** **Specifications**					**5. Outcome Measures Brain**			**6. Outcome Measures Behavior**		**7. Data Storage**
	**1a**	**1b**	**2a**	**2b**	**2c**	**2d**	**2e**	**3a**	**3b**	**3c**	**3d**	**3e**	**4a**	**4b**	**4c**	**4d**	**4e**	**5ª**	**5b**	**5c**	**6a**	**6b**	**7a**
Domingos et al. (2021)	-	2	2	-	-	-	2	2	2	3	-	-	-	2	2	2	2	5	3	3	5	7	8
Domingos et al. (2020)	-	-	10	-	-	-	10	9	10	10	-	-	-	11	10	10	10	12	12	12	-	14	-
Mikicin et al. (2015)	-	-	435	-	-	-	435	-	435	435	-	-	-	436	436	436	436	438/441	442/443	438/439	-	441	-
Mikicin et al. (2018)	-	74	74	-	-	-	74	-	-	-	-	-	-	77	73	-	-	77	74	76	77	76/77	-
Parsaee et al. (2018)	-	-	4	-	-	-	4	-	4	4	-	-	-	4	4	4	4	4	4	4	-	4/5	-
Paul et al. (2011)	-	-	33	-	-	-	33	33	33	33/34	-	-	-	33	33	34	33	36	36	34/36	-	36	-
Rostami et al. (2012)	-	-	265	-	-	-	265	265	265	265	-	-	-	265	265	265	266	266	266	266	-	266	-
Salimnejad et al. (2019)	-	13	13	-	-	-	13	-	14	14	-	-	-	14	14	14	14	16	15	14	-	15	-

*1. Pre-experiment (1a. Preregister experimental protocol and planned analyses; 1b. Justify sample size); 2. Control groups (2a. Employ control group(s) or control condition(s); 2b. When leveraging experimental designs where a double-blind is possible, use a double-blind; 2c. Blind those who rate the outcomes, and when possible, the statisticians involved; 2d. Examine to what extent participants and experimenters remain blinded; 2e. In clinical efficacy studies, employ a standard-of-care intervention group as a benchmark for improvement); 3. Control measures (3a. Collect data on psychosocial factors; 3b. Report whether participants were provided with a strategy; 3c. Report the strategies participants used; 3d. Report methods used for online data processing and artifact correction; 3e. Report condition and group effects for artifacts); 4. Feedback specifications (4a. Report how the online feature extraction was defined; 4b. Report and justify the reinforcement schedule; 4c. Report the feedback modality and content; 4d. Collect and report all brain activity variable(s) and/or contrasts used for feedback, as displayed to experimental participants; 4e. Report the hardware and software used); 5. Outcome measures Brain (5a. Report neurofeedback regulation success based on the feedback signal; 5b. Plot within-session and between-session regulation blocks of feedback variable(s), as well as pre-to-post resting baselines or contrasts; 5c. Statistically compare the experimental condition/group to the control condition(s)/group(s) (not only each group to baseline measures)); 6. Outcome measures Behavior (6a. Include measures of clinical or behavioral significance, defined a priori, and describe whether they were reached; 6b. Run correlational analyses between regulation success and behavioral outcomes); 7. Data Storage (7a. Upload all materials, analysis scripts, code, and raw data used for analyses, as well as final values, to an open access data repository, when feasible)*.

**Table 2:** Critical appraisal assessment checklist for randomized controlled trials.

**References**	**Scale**	**1**	**2**	**3**	**4**	**5**	**6**	**7**	**8**	**9**	**10**	**11**	**12**	**13**
Domingos et al. (2021)	Yes	x		X	x	X	x	x		x	x	x	x	X
	Not		x						X					
	Unclear													
	Not applicable													
Domingos et al. (2020)	Yes	x		X	x	X	x	x		x	x	x	x	X
	Not		x						X					
	Unclear													
	Not applicable													
Mikicin et al. (2015)	Yes	x			x	X		x	X	x	x	x	x	X
	Not		x	X			x							
	Unclear									x	x	x	x	X
	Not applicable													
Mikicin et al. (2018)	Yes			X				x	X	x	x	x	x	
	Not													
	Unclear		x											X
	Not applicable	x			x	x	x							
Paul et al. (2012)	Yes	x		X				x	X	x	x	x	x	X
	Not		x				x							
	Unclear				x	X								
	Not applicable													
Rostami et al. (2012)	Yes			X					X	x	x	x	X	X
	Not	x												
	Unclear		x		x	X	x	x						
	Not applicable													
Salimnejad et al. (2019)	Yes	x						x		x	x	x	X	X
	Not													
	Unclear		x		x	X	x		X					
	Not applicable			X										
Parsaee et al. (2018)	Yes	x		X				x		x	x	x	X	X
	Not		x											
	Unclear				x	X	x		X					
	Not applicable													

*1. Was true randomization used for assignment of participants to treatment groups?; 2. Was allocation to treatment groups concealed?; 3. Were treatment groups similar at the baseline?; 4. Were participants blind to treatment assignment?; 5. Were those delivering treatment blind to treatment assignment?; 6. Were outcomes assessors blind to treatment assignment?; 7. Were treatment groups treated identically other than the intervention of interest?; 8. Was follow up complete and if not, were differences between groups in terms of their follow up adequately described and analyzed?; 9. Were participants analyzed in the groups to which they were randomized?; 10. Were outcomes measured in the same way for treatment groups?; 11. Were outcomes measured in a reliable way?; 12. Was appropriate statistical analysis used?; 13. Was the trial design appropriate, and any deviations from the standard RCT design (individual randomization, parallel groups) accounted for in the conduct and analysis of the trial?*

### Inclusion Criteria

Articles in English were selected as follows: primary studies; young people and adults (16–30 years old), healthy, practicing sports; intervention using the NFB; studies with at least two groups or pre- and post-intervention evaluation; and outcome with indices of reaction time and cognitive skills.

### Exclusion Criteria

Regarding exclusion criteria, we adopted studies that did not present sufficient information about the type of intervention and incomplete statistical data and participants with chronic diseases or medications that affect executive functions.

This study used the search strategy described, obtaining 1,530 articles in the databases. Of this total, 285 duplicates were identified, and titles and abstracts excluded 1,225. Of the 20 remaining articles, only 10 met the final eligibility criteria according to PRISMA, as shown in Figure 1.

### Quality Assessment

The evaluation and discussion of the quality of the articles were carried out independently, and by peers, for the quality of the included studies, all data were combined using Review Manager 5.3 (http://tech.cochrane.org/revman/download) and we used the risk of bias tool developed by The Cochrane Collaboration (Higgins et al., 2019). We also included experimental study characteristics and methodological quality according to JBI classifications (Peters et al., 2020) and design and reporting quality according to the CRED-nf checklist (Ros et al., 2020).

### Quantitative Analysis

The Kappa coefficient test was applied to assess the agreement and reliability of studies between authors. We applied the values of 0–0.20, no agreement between raters, 0.21–0.39, minimum agreement, 0.40–0.59, weak agreement, 0.60–0.79, moderate agreement, 0.80–0.90, strong agreement, and >90 almost perfect (McHugh, 2012).

The variables measured were reaction time and cognitive performance indices. We used continuous data of M, SD, and the number of participants (*n*). We applied the inverse variance method and the random-effects analysis model. We calculated a standardized mean difference (SMD) with 95% confidence intervals (CIs) for each study, defined as the difference in mean post-treatment changes between the two groups (NFB Group vs. Control Groups).

Using the random-effects models (Field and Gillett, 2010), we interpreted the SMDs using 0.2 to represent a small effect, 0.5 to represent a moderate effect, and 0.8 to represent a large effect (Cohen et al., 1988).

For the heterogeneity of the studies, the *I*^2^ statistic was applied, a quantitative measure of inconsistency between the studies. Studies with an *I*^2^ statistic of 25–50% were considered low heterogeneity, an *I*^2^ statistic of 50–75% were considered moderate heterogeneity, and an *I*^2^ statistic > 75% were considered high heterogeneity (Borenstein et al., 2017; Ruppar, 2020). All data were combined using Review Manager 5.4 (https://training.cochrane.org/online-learning/core-software-cochrane-reviews/revman).

## Results

### Consensus on the Reporting and Experimental Design of Clinical and Cognitive-Behavioral NFB Studies (CRED-NF)

The assessment as the CRED-NF (Ros et al., 2020) was performed in all articles included in the study. It aims to present a consensus checklist that aims to improve study standards. The questions were divided into six control parameters, namely, pre-experiment, control groups, control measures, feedback specifications, outcome measures, whether cerebral or behavioral data, composed of specific questions for each parameter. The agreement between raters was 93%. The data are shown in Table 1.

### Critical Appraisal Checklist for Randomized Controlled Trials (JBI)

Qualitative analysis was performed using the JBI classifications (Peters et al., 2020), which aims to assess the methodological quality of the studies and determine the extent to which a study addressed the possibility of bias in its design, conduct, and analysis by answering yes, no, unclear, or not applicable. It was verified whether the protocols used in the articles met the eligibility criteria. The agreement between raters was 90%. The assessments are illustrated in Table 2.

### Assessment of Study Bias Risk

**Figure 2:**
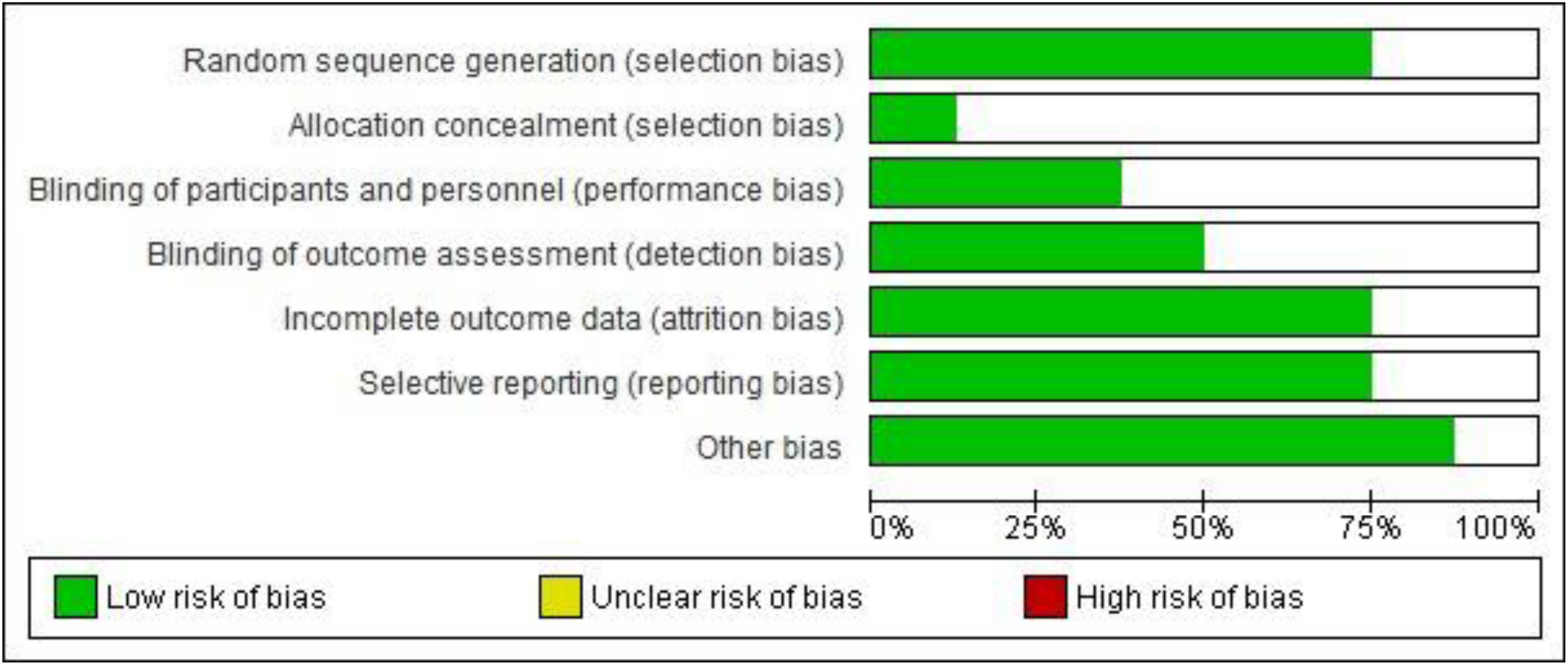
Risk of bias graph: review the authors' judgments on each risk of bias item presented as percentages across all included studies. Created using Software Review Manager 5.4.1.

The studies presented an adequate assessment regarding the low risk of bias. Regarding the random generation sequence to select participants, the studies showed a 75% low risk of bias, but only one study reported the allocation methods in detail. Included studies were rated unclear in the domains of blinding techniques of participants and assessment. Regarding information on data, more than 80% had a low risk of bias. The reliability agreement between rates was 98%. They are indicated in Figure 2.

Table 3 shows the characteristics of the studies included in the meta-analysis, reaction time, and NFB effects in sports.

**Table 3:** Characteristics of the studies included in the meta-analysis-reaction time and NFB.

**References**	**Study design**	**Objective**	**Sport**	**Age (M)**	**Experience (DP)**	**Intervention**	**Test**	**Type of NFB**	**Trained brain waves**	**Trained brain area**	**Cognitive training**	**Outcome**
Mikicin et al. (2015)	Pre-experimental study	Evaluate the impact of such holistic training on physiological (EEG) and behavioral measures on semi-professional athletes.	Swimming, fencing, athletics, taekwondo and judo	18 a 25	5 a 7	Experimental Group (*n* = 25): 20 sessions, for 4 months	Attention and reaction	NFB–EEG	↓Beta 2 (20–30 Hz), ↓Teta (4–7.5 Hz) e ↑Beta 1 (13–20 Hz), ↑SMR (12–15 Hz)	C3 e C4	Attention and concentration	↓ reaction time
						Control Group (*n* = 10)						
Parsaee et al. (2018)	Pre-experimental study	Investigate the effect of neurofeedback training on the visual and auditory reaction time of veterans and disabled athletes.	Sports shooting	> 30	5	Experimental Group (*n* = 8): 15 sessions, 3x a week, duration 30'	Tests Strop	NFB–EEG	↓Theta (4–6 Hz) e ↑SMR (12–15 Hz)	Cz	Speed and efficiency of decision making	Improving brain functions for ↓reaction time in visual simple, auditory simple, visual selective, and auditory selectivity
						Control Group (*n* = 8)						
Mikicin et al. (2018)	Pre-experimental study	Analyse changes in the level of attention and activation in sports shooters after neurofeedback-EEG training.	Sports shooting	19 a 21	–	Experimental Group (*n* = 17): 20 sessions 1/2 x a week, duration de 40'	Test COG e FLIM	NFB–EEG	↑Beta (12–22 Hz)	F3, F4, P3 e P4	General attention	↑ attention level, quickly and accurately
						Control Group (*n* = 10)						
Rostami et al. (2012)	Pre-experimental study	Compare rifle shooters' performance between two groups of expert shooters, one trained with a neurofeedback method and the other not trained.	Sports shooting	30.0 ± 6.7	7.5 ± 6.13	Experimental Group (*n* = 12): 15 sessions, 3x a week, duration 60'	Performance indicators	NFB–EEG	1ªEtapa: ↑SMR (13–15 Hz), ↓Beta (20–30 Hz), 2ª etapa: Alfha (8–12 Hz), Theta (4–8 Hz) e ↑Beta (20–30 Hz)	C3, C4 e Pz	Stability, accuracy and reaction time	Improve rifle shooters' performances, ↓aiming time ↓
				30.92 ± 5.52	6.58 ± 4.87	Control Group (*n* = 12)						

*SMR, sensorimotor rhythm. ↓, ↑ means increase and decrease respectively*.

Table 4 demonstrates the features of the studies included in the meta-analysis, cognitive performance abilities, and NFB results between athletes.

**Table 4:** Characteristics of the studies included in the meta-analysis—cognitive performance and NFB.

**References**	**Study design**	**Objective**	**Sport**	**Age (M)**	**Experience (DP)**	**Intervention**	**Test**	**Type of NFB**	**Trained brain waves**	**Trained brain area**	**Cognitive training**	**Outcome**
Domingos et al. (2021)	Pre-experimental study	Investigation was to understand if there are differences between performing two sessions or three sessions per week in enhancement of alpha activity and improvement of cognition	Federated athletes (M)	22.60 ± 1.12	5	Experimental Group (*n* = 15): 12 sessions, 3x a week, duration 25'	Digit Span e Oddball	NFB–EEG	Alpha (IAB: 4-30 Hz)	–	Attention and memory	↑ Cognitive performance
				22.53 ± 3.89		Control Group (*n* = 15)						
Domingos et al. (2020)	Pre-experimental study	Neurofeedback training protocol implemented in a nonathletic population can improve short-term memory and reaction time in athletes	Federated athletes (M)	27.93 ± 6.11	5	Experimental Group (*n* = 15): 12 sessions, 2 x a week, duration 25'	Digit Span, N-Back e Oddball	NFB–EEG	Alpha (IAB: 4-30 Hz)	–	Reaction time	↑ Cognitive performance
				22.53 ± 3.89		Control Group (*n* = 15)						
Paul et al. (2011)	Pre-experimental study	find out the effect of neurofeedback training on Improvement of the archery performance.	Archery and Fleet Athletes (16Me 8F)	21.96 ± 1.60	4.31 ± 1.08	Experimental Group (*n* = 12) 12 sessions, 3x a week, duration de 20'	Performance level	NFB–EEG	↑SMR (12–15 Hz), ↓Theta (4–7 Hz) e ↑Beta (22–26 Hz)	Cz	Excitement, performance, control and precision	↑ Performance
						Control Group (*n* = 12)						
Rostami et al. (2012)	Pre-experimental study	Compare rifle shooters' performance between two groups of expert shooters, one trained with a neurofeedback method and the other not trained.	Sports shooting	30.0 ± 6.7	7.5 ± 6.13	Experimental Group (*n* = 12): 15 sessions, 3x a week, duration 60'	Sniper Performance Measurs	NFB–EEG	1ªEtapa: ↑SMR (13–15 Hz), ↓Beta (20–30 Hz), 2ª etapa: Alfha (8–12 Hz), Theta (4–8 Hz) e ↑Beta (20–30 Hz	C3, C4 e Pz	Stability, accuracy and reaction time	↑Cognitive Performance
				30.92 ± 5.52	6.58 ± 4.87	Control Group (*n* = 12)						
Salimnejad et al. (2019)	Pre-experimental study	Determine the effect of bio-neural feedback exercises on female rugby players' performance.	Rugby (F)	16 a 25	–	Experimental Group (*n* = 12): 15 sessions, 3x a week; duration de 40'	Accuracy of the shot	NFB–EEG	Alpha e ↑SMR	Pz e C3	Precision	↑Cognitive Performance
						Control Group (*n* = 12)						

*↓, ↑ means increase and decrease respectively*.

**Figure 3:**
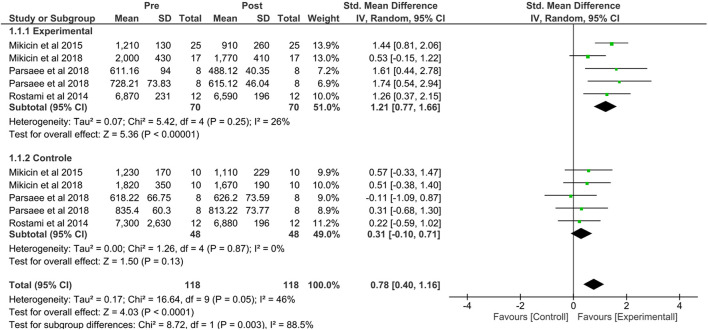
Forest plot comparing the NFB group with the control group after sports performance indicating the improvement in the reaction time of the athletes. Created using Software Review Manager 5.4.1.

The studies that evaluated athletes' reaction time comparing the NFB group with the control group are shown in Figure 3.

The experimental group showed a large effect [SMD = 1.21, 95% CI (0.77, 1.66), *p* = 0.00001], indicating that athletes who participated in training with the NFT on average had a more significant reduction in reaction time than athletes in control conditions. An *I*^2^ of 26% suggested a low level of heterogeneity among these studies.

When comparing the pre- and post-intervention groups, the NFB group had a high effect size [*Z* = 4.03; SMD = 0.78; 95% CI = (0.40, 1.16), *p* = 0.00001], indicating that the athletes who participated in the NFB training on average had a significantly shorter reaction time than athletes in the post-intervention control group. Regarding heterogeneity, the *I*^2^ of 88.5% suggested a substantial heterogeneity between these studies.

**Figure 4:**
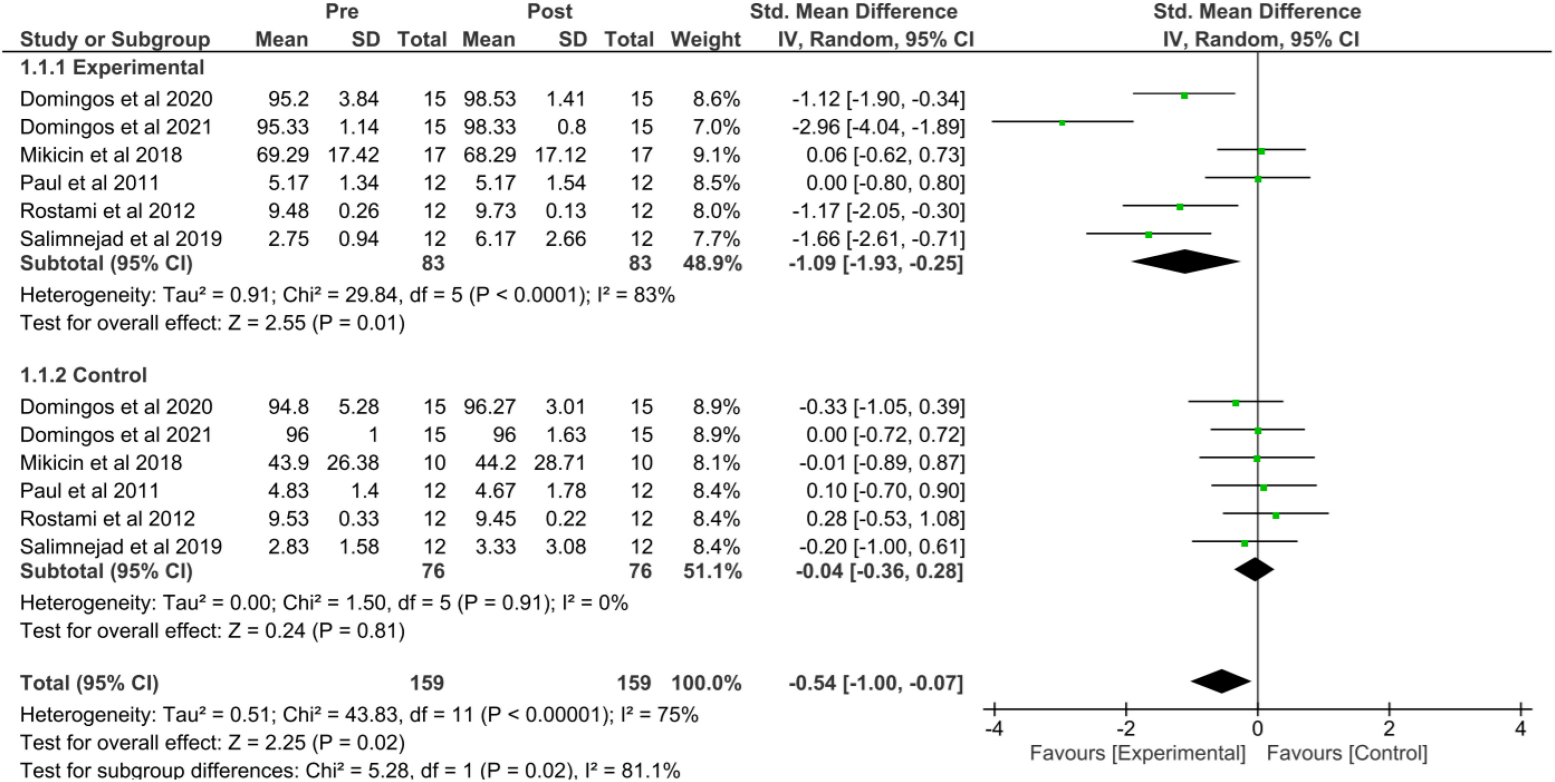
Forest plot comparing the NFB and control groups with the pre and post-performance. Created using Software Review Manager 5.4.1.

The studies that evaluated cognitive performance, comparing the NFB group and the control group, are shown in Figure 4.

The experimental group showed a large beneficial effect on sports performance [SMD = −1.09, 95% CI = (−1.93, −0.25), *p* = 0.0001], indicating that athletes who participated in training with the NFT on average had the significantly higher cognitive performance than athletes under control conditions. An *I*^2^ of 83% suggested a high level of heterogeneity among these studies.

When comparing the pre- and post-intervention groups, the NFB group had a high effect size [*Z* = 2.25; SMD = −0.54; 95% CI = (−1.00, 0.07), *p* = 0.02], indicating that athletes who participated in training with NFB on average had significantly higher cognitive performance than athletes in the post-intervention control group. Regarding heterogeneity, the *I*^2^ of 81.1% suggested a substantial heterogeneity between these studies.

**Figure 5:**
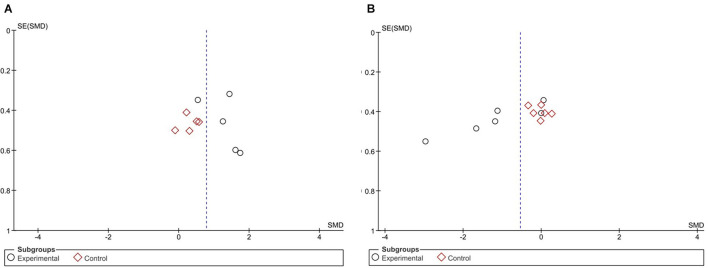
**(A,B)** Risk of bias from studies on the effect of NFB on reaction time on sports performance (Figure 4A) and cognitive skills (Figure 4B). Created using Software Review Manager 5.4.1.

Figure 5A shows the risk of bias for the studies included in the meta-analysis of Figure 3, while Figure 5B shows the risk of bias for the studies shown in the forest plot of Figure 4.

## Discussion

This study aimed to evaluate the effect of training with NFB on improving athletes' reaction time and cognitive performance. The results of this meta-analysis indicated that training with NFB is practical for self-regulation of athletes, concerning an improvement in reaction time, with a large effect (5.36) [SMD = 1.21, 95% CI = (0.77, 1.66), *p* = 0.00001], as well as on cognitive performance, showed a large effect (2.55) [SMD = −1.09, 95% CI = (−1.93, −0.25), *p* = 0.0001], indicating that athletes who participated in the training with the NFT performed significantly better than athletes under control conditions.

The effect size of this study indicated that training with NFB can increase the performance of athletes, using reaction time and cognitive performance as an evaluative index. As for the reaction time, we observed that the athletes who participated in the training with NFB showed sports performance significantly higher than the athletes in the control group after the intervention. The reaction time of the athletes decreased, indicating training effectiveness. The results indicated a significant improvement in reaction time. The studies that evaluated cognitive performance indicated an improvement in the NFB group compared to the control group, pre-, and post-intervention. The performance was higher post-intervention, recommending the effectiveness of training with NFB.

About the studies that evaluated the reaction time, Mikicin et al. (2015) used the EEG-NFB for the amplification of the sensorimotor rhythm (SMR) (12–15 Hz) and beta1 (13–20 Hz) bands and simultaneous reduction of theta (4–7.5 Hz) and beta2 (20–30 Hz). The trained group exhibited more significant reductions in reaction times on a test of visual attention than the control group and showed an improvement in several performance measures used to assess speed, effectiveness, and accuracy of work. Furthermore, Mikicin et al. (2018) performed NFB-EEG training to strengthen the beta frequency (12–22 Hz). The differences between the first and second measures show that the shooters included in the study improved their attention-level skills. Subjects performed the task more quickly and accurately during the second measurement. Parsaee et al. (2018) performed a protocol of increasing SMR and theta decline. The results demonstrated that NFB training reduced the reaction time in simple visual, simple auditory, selective visual, and auditory selectivity. All studies have concluded that NFB training effectively improves brain functions for a reaction time in athletes.

Regarding the studies that evaluated cognitive performance, Domingos et al. (2020) indicated that training with NFB increases the relative amplitude of the bands in the group of nonathletes; however, only athletes were shown to improve performance tests after 12 sessions of NFB training. Domingos et al. (2021) showed that NFB training with three sessions per week was more effective in increasing alpha amplitude during NFB training than two sessions per week. Furthermore, only the three sessions per week group showed a significant improvement in performance after training. The results suggest that condensed training protocols lead to better results, guiding NFB protocol design to optimize training effectiveness. According to Paul et al. (2011), NFB training improves the regularity of archery players in scoring, increasing the accuracy of the arrow shot obtained by the control and regulation of psychophysiological and electroencephalographic measures. Rostami et al. (2012) concluded that the NFB could be suggested to improve the performance of rifle shooters. Salimnejad et al. (2019) training with NFB can be used as an effective way to improve the optimal performance of athletes in sports, such as rugby, which require precise passes.

As described above, all studies indicated positive results with NFB training, despite heterogeneity with subjects, sports (e.g., swimming, fencing, athletics, taekwondo, judo, sport shooting, archery, and rugby), treatment conditions, and about EEG bands (e.g., SMR, alpha, beta, and theta). There was a variety of duration and frequency of training with NFB, not finding a standard protocol among the studies. For example, most studies have selected different frequency bands and different locations to measure the effects of NFB.

In the studies, the SMR, alpha, and beta bands were the most effective in improving sports performance (Raymond et al., 2005; Cherapkina, 2012; Gruzelier, 2014; Cheng et al., 2015). An increase in SMR is associated with an increase in attention (Cheng et al., 2015), which corroborates the chosen evaluative indices, reaction time, and performance.

## Conclusion

Our findings demonstrated that training with NFB in EEG power presents a more significant reduction in reaction time and improvement in the cognitive performance of athletes, effectively increasing their performance. According to the studies presented, we suggest a training protocol with NFB to reduce reaction time, with 18 training sessions lasting 45 min, training in beta (12–15 Hz), SMR (12–15 Hz), and theta waves (4–8 Hz), for C3, C4, Cz, F3, F4, P3, P4, and Pz areas. For cognitive performance training, we propose 15 NFB sessions, lasting 37 min, in alpha (4–8 Hz), beta (12–30 Hz), SMR (12–15 Hz), and theta (4–8 Hz) waves, for C3, C4, Cz, F3, F4, P3, P4, and Pz areas, considering the mental mapping of athletes. Future research on reaction time and cognitive performance in sports can use the results of this systematic review with meta-analysis as a guide for developing protocols and improving the control and manipulation of NFB interventions.

## Data Availability Statement

The original contributions presented in the study are included in the article/supplementary material, further inquiries can be directed to the corresponding author/s.

## Author Contributions

MB, JF, NE, DA, and BM participated in the study design, search and selection of articles, collection, data analysis, and manuscript preparation. DV and MS participated in the data analysis and manuscript preparation. NB and CB participated in the manuscript review. All authors contributed to the article and approved the submitted version.

## Conflict of Interest

The authors declare that the research was conducted in the absence of any commercial or financial relationships that could be construed as a potential conflict of interest.

## Publisher's Note

All claims expressed in this article are solely those of the authors and do not necessarily represent those of their affiliated organizations, or those of the publisher, the editors and the reviewers. Any product that may be evaluated in this article, or claim that may be made by its manufacturer, is not guaranteed or endorsed by the publisher.

## References

[B1] Araújo D. Hristovski R. Seifert L. Carvalho J. Davids K. (2019). Ecological cognition: expert decision-making behaviour in sport. Int. Rev. Sport Exerc. Psychol. 12:1–25. 10.1080/1750984X.2017.134982632962508

[B2] Berwanger O. Suzumura E. A. Buehler A. M. Oliveira J. B. (2007). Como avaliar criticamente revisões sistemáticas e metanálises? Revista Brasileira de Terapia Intensiva 19:475–480. 10.1590/S0103-507X2007000400012 25310166

[B3] Blumenstein B. Orbach I. (2020). Periodization of psychological preparation within the training process. Int. J. Sport Exerc. Psychol. 18:13–23. 10.1080/1612197X.2018.1478872

[B4] Borenstein M. Higgins J. P. Hedges L. V. Rothstein H. R. (2017). Basics of meta-analysis: I2 is not an absolute measure of heterogeneity. Res. Synth. Method. 8:5–18. 10.1002/jrsm.1230 28058794

[B5] Bramer W. M. Milic M. D. J Mast F. Ph,D, (2017). Reviewing retrieved references for inclusion in systematic reviews using EndNote. J. Med. Libr. Assoc. 105:111. 10.5195/jmla.2017.111 28096751 PMC5234463

[B6] Cheng M.-Y. Huang C.-J. Chang Y.-K. Koester D. Schack T. Hung T.-M. (2015). Sensorimotor rhythm NFB enhances golf putting performance. J. Sport Exerc. Psychol. 37:626–636. 10.1123/jsep.2015-0166 26866770

[B7] Cherapkina L. (2012). The NFB successfulness of sportsmen. J. Hum. Sport Exerc. 7, S116–S127. 10.4100/jhse.2012.7.Proc1.13

[B8] Cohen Z. Vonshak A. Richmond A. (1988). Effect of environmental conditions on fatty acid composition of the red alga Porphyridium cruentum: Correlation to growth rate 1. J. Phycol. 24, 328–332.

[B9] Crivelli D. Fronda G. Balconi M. (2019). Neurocognitive enhancement effects of combined mindfulness–NFB training in sport. Neuroscience 412:83–93. 10.1016/j.neuroscience.2019.05.066 31195055

[B10] Diamond A. (2013). Executive functions. Ann. Rev. Psychol. 64:135–168. 10.1146/annurev-psych-113011-143750 23020641 PMC4084861

[B11] Diamond A. Ling D. S. (2016). Conclusions about interventions, programs, and approaches for improving executive functions that appear justified and those that, despite much hype, do not. Dev. Cogn. Neurosci. 18:34–48. 10.1016/j.dcn.2015.11.005 26749076 PMC5108631

[B12] Domingos C. Alves C. Sousa E. Rosa A. Pereira J. (2020). Does NFB training improve performance in athletes? NeuroRegulation 7:8–17. 10.15540/nr.7.1.8

[B13] Domingos C. Peralta M. Prazeres P. Nan W. Rosa A. Pereira J. G. (2021). Session frequency matters in neurofeedback training of athletes. Appl. Psychophysiol. Biofeedback 46:195–204. 10.1007/s10484-021-09505-3 33528679

[B14] Field A. P. Gillett R. (2010). How to do a meta-analysis. Br. J. Mathemat. Statist. Psychol. 63, 665–694. 10.1348/000711010X502733 20497626

[B15] Filho E. Dobersek U. Husselman T.-A. (2021). The role of neural efficiency, transient hypofrontality and neural proficiency in optimal performance in self-paced sports: a meta-analytic review. Exp. Brain Res. 239:1381–1393. 10.1007/s00221-021-06078-9 33760959

[B16] Gomes V. R. I. Rêgo C. O. D. M. (2019). Guia de orientação para o desenvolvimento de habilidades psicológicas na reabilitação de atletas de alto rendimento.

[B17] Gong A. Gu F. Nan W. Qu Y. Jiang C. Fu Y. (2021). A review of NFB training for improving sport performance from the perspective of user experience. Front. Neurosci. 15:638369. 10.3389/fnins.2021.638369 34127921 PMC8195869

[B18] Gruzelier J. H. (2014). EEG-NFB for optimising performance. III: a review of methodological and theoretical considerations. Neurosci. Biobehav. Rev. 44:159–182. 10.1016/j.neubiorev.2014.03.015 24690579

[B19] Higgins J. P. Thomas J. Chandler J. Cumpston M. Li T. Page M. J. . (2019). Cochrane Handbook for Systematic Reviews of Interventions. John Wiley and Sons.

[B20] Liu Y. Subramaniam S. C. H. Sourina O. Shah E. Chua J. Ivanov K. (2017). “NFB training for rifle shooters to improve cognitive ability,” in 2017 International Conference on Cyberworlds (Singapore), 186–189. 10.1109/CW.2017.36

[B21] McHugh M. L. (2012). Interrater reliability: The kappa statistic. Biochemia medica. 22, 276–282. 23092060 PMC3900052

[B22] Mikicin M. Orzechowski G. Jurewicz K. Paluch K. Kowalczyk M. Wróbel A. (2015). Brain-training for physical performance: a study of EEG-NFB and alpha relaxation training in athletes. Acta Neurobiol. Exp. 75, 434–445. 26994421

[B23] Mikicin M. Szczypinska M. Skwarek K. (2018). NFB needs support! Effects of NFB-EEG training in terms of the level of attention and arousal control in sports shooters. Baltic J. Health Phys. Act. 10:72–79. 10.29359/BJHPA.10.3.08

[B24] Mirifar A. Beckmann J. Ehrlenspiel F. (2017). NFB as supplementary training for optimizing athletes' performance: a systematic review with implications for future research. Neurosci. Biobehav. Rev. 75:419–432. 10.1016/j.neubiorev.2017.02.005 28185873

[B25] Mirifar A. Keil A. Beckmann J. Ehrlenspiel F. (2019). No effects of NFB of beta band components on reaction time performance. J. Cogn. Enhanc. 3:251–260. 10.1007/s41465-018-0093-0

[B26] Ouzzani M. Hammady H. Fedorowicz Z. Elmagarmid A. (2016). Rayyan—a web and mobile app for systematic reviews. Systemat. Rev. 5:210. 10.1186/s13643-016-0384-4 27919275 PMC5139140

[B27] Page M. J. Shamseer L. Tricco A. C. (2018). Registration of systematic reviews in PROSPERO: 30,000 records and counting. Systemat. Rev. 7:32. 10.1186/s13643-018-0699-4 29463298 PMC5819709

[B28] Paret C. Goldway N. Zich C. Keynan J. N. Hendler T. Linden D. . (2019). Current progress in real-time functional magnetic resonance-based NFB: methodological challenges and achievements. NeuroImage 202:116107. 10.1016/j.neuroimage.2019.116107 31437551

[B29] Parsaee S. Alboghbish S. Abdolahi H. Alirajabi R. Anbari A. (2018). Effect of a period of selected SMR/Theta NFB training on visual and auditory reaction time in veterans and disabled athletes. Iran. J. War Public Health 10:15–20. 10.29252/ijwph.10.1.15

[B30] Paul M. Ganesan S. Sandhu J. S. Simon J. V. (2012). Effect of sensory motor rhythm neurofeedback on psycho-physiological, electro-encephalographic measures and performance of archery players. Ibnosina J. Med. Biomed. Sci. 4, 32–39. 10.4103/1947-489X.210753

[B31] Peters M. Godfrey C. McInerney P. Munn Z. Trico A. Khalil H. (2020). “ Scoping reviews,” in JBI Manual for Evidence Synthesis, eds E. Aromataris and Munn JBI (Sydney, NSW). 10.46658/JBIMES-20-12

[B32] Raymond J. Varney C. Parkinson L. A. Gruzelier J. H. (2005). The effects of alpha/theta NFB on personality and mood. Cogn. Brain Res. 23:287–292. 10.1016/j.cogbrainres.2004.10.023 15820636

[B33] Ros T. Enriquez-Geppert S. Zotev V. Young K. D. Wood G. Whitfield-Gabrieli S. . (2020). Consensus on the reporting and experimental design of clinical and cognitive-behavioural NFB studies (CRED-nf checklist). Brain 143:1674–1685. 10.1093/brain/awaa009 32176800 PMC7296848

[B34] Rostami R. Sadeghi H. Karami K. A. Abadi M. N. Salamati P. (2012). The effects of NFB on the improvement of rifle shooters' performance. J. Neurother. 16, 264–269. 10.1080/10874208.2012.730388

[B35] Ruppar T. (2020). Meta-analysis: how to quantify and explain heterogeneity? Eur. J. Cardiovasc. Nurs. 19:646–652. 10.1177/1474515120944014 32757621

[B36] Russo G. Ottoboni G. (2019). The perceptual – cognitive skills of combat sports athletes: a systematic review. Psychol. Sport Exerc. 44:60–78. 10.1016/j.psychsport.2019.05.004

[B37] Salimnejad Z. Zandi H. Arsham S. (2019). Effect of bio-neural feedback exercises on the performance of female Rugby players. Int. J. Motor Control Learn. 1:10–18. 10.29252/ijmcl.1.2.10

[B38] Schönenberg M. Wiedemann E. Schneidt A. Scheeff J. Logemann A. Keune P. M. . (2017). NFB, sham NFB, and cognitive-behavioural group therapy in adults with attention-deficit hyperactivity disorder: a triple-blind, randomised, controlled trial. Lancet Psychiatr. 4:673–684. 10.1016/S2215-0366(17)30291-2 28803030

[B39] Sitaram R. Ros T. Stoeckel L. Haller S. Scharnowski F. Lewis-Peacock J. . (2017). Closed-loop brain training: the science of NFB. Nat. Rev. Neurosci. 18:86–100. 10.1038/nrn.2016.164 28003656

[B40] Tricco A. C. Lillie E. Zarin W. O'Brien K. K. Colquhoun H. Levac D. . (2018). PRISMA extension for scoping reviews (PRISMA-ScR): checklist and explanation. Ann. Internal Med. 169:467–473. 10.7326/M18-0850 30178033

[B41] Xiang M.-Q. Hou X.-H. Liao B.-G. Liao J.-W. Hu M. (2018). The effect of NFB training for sport performance in athletes: a meta-analysis. Psychol. Sport Exerc. 36:114–122. 10.1016/j.psychsport.2018.02.004

